# Thermally Switchable Electrically Conductive Thermoset rGO/PK Self-Healing Composites

**DOI:** 10.3390/polym13030339

**Published:** 2021-01-21

**Authors:** Esteban Araya-Hermosilla, Alice Giannetti, Guilherme Macedo R. Lima, Felipe Orozco, Francesco Picchioni, Virgilio Mattoli, Ranjita K. Bose, Andrea Pucci

**Affiliations:** 1 Center for Micro-BioRobotics, Istituto Italiano di Tecnologia, Viale Rinaldo Piaggio 34, Pontedera, 56025 Pisa, Italy; esteban.araya@iit.it (E.A.-H.); virgilio.mattoli@iit.it (V.M.); 2 Dipartimento di Chimica e Chimica Industriale, Università di Pisa, Via Moruzzi 13, 56124 Pisa, Italy; alice.giannetti@hotmail.it; 3 Department of Chemical Product Engineering, Engineering and Technology Institute Groningen (ENTEG), University of Groningen, Nijenborgh 4, 9747AG Groningen, The Netherlands; g.de.macedo.rooweder.lima@rug.nl (G.M.R.L.); f.orozco.gutierrez@rug.nl (F.O.); f.picchioni@rug.nl (F.P.); 4 Centro per l’Integrazione della Strumentazione dell’Università di Pisa (CISUP), Lungarno Pacinotti 43, 56126 Pisa, Italy

**Keywords:** self-healing, nanocomposite, reduced graphene oxide, smart polymer, polyketone, electrical conductivity

## Abstract

Among smart materials, self-healing is one of the most studied properties. A self-healing polymer can repair the cracks that occurred in the structure of the material. Polyketones, which are high-performance thermoplastic polymers, are a suitable material for a self-healing mechanism: a furanic pendant moiety can be introduced into the backbone and used as a diene for a temperature reversible Diels-Alder reaction with bismaleimide. The Diels-Alder adduct is formed at around 50 °C and broken at about 120 °C, giving an intrinsic, stimuli-responsive self-healing material triggered by temperature variations. Also, reduced graphene oxide (rGO) is added to the polymer matrix (1.6–7 wt%), giving a reversible OFF-ON electrically conductive polymer network. Remarkably, the electrical conductivity is activated when reaching temperatures higher than 100 °C, thus suggesting applications as electronic switches based on self-healing soft devices.

## 1. Introduction

Smart polymers are a category of materials able to change their features in response to the changing characteristics of the external environment [1]. A stimulus, such as a mechanical stress, a change in temperature or pH, is sufficient to easily modify the structure of the material [2], thus suggesting useful applications in various fields. A change in the structure of the material triggered by external stimuli is for example used to produce carriers for drug delivery [3]. On this account, Convertine et al. developed a diblock copolymer with a temperature-responsive micellization [4], whereas Mahmoodzadeh et al. reported a pH-sensitive triblock copolymer as a drug delivery system for cancer therapy [5]. Hajebi et al. proposed a temperature and pH-responsive nanogel for the controlled release of doxorubicin [6]. Furthermore, smart polymers are used to fabricate high-performance sensors [7], in chromatography [8] and in the oil industry [9]. Nowadays, smart materials are also endowed with certain self-healing characteristics [10], that are fully inspired by the typical mechanisms of the biological world [11]. Notably, local and temporary mobility must be achieved to get an effective self-healing material but without hindering the structural application of the whole material. Only the damaged part should be involved in the self-healing process. For this reason, local healing is provided by heating or irradiating a small portion of the broken material, while the bulk structure can retain the overall toughness [12]. The stimulus can be also provided by electrical current, that is able to repair the material’s damage thanks to the Joule effect [13]. The healing stimulus can be switched ON and OFF quickly depending on the kinetics of the healing mechanism and the chemical groups involved in the process [14]. From the above motivations, our group has extensively worked on self-healing thermosets based on the reversible Diels-Alder reaction [15,16,17,18]. From the perspective to produce scalable thermoset systems, our research has been focusing on the chemical modification of polyketone with primary amines via the Paal-Knorr reaction since it is a feasible route to produce these materials at an industrial level [19]. This synthetic approach provides several advantages; it can be carried out in bulk, without catalysts, with high yields at relatively mild conditions, and with water being the only by-product [19]. In addition, it can be also carried out without any solvent or in various organic solvents depending on the physical and chemical properties of the amine compound [20]. A variety of primary amines can be used, making this synthesis a fast, cheap, and appealing approach to create polymers with almost any desired pendant functional group [21,22]. Self-healing thermosets have been efficiently prepared by modifying polyketone with furan groups which can undergo thermally reversible Diels-Alder reactions with maleimides compounds [16,17,23,24]. Diels-Alder adducts can be easily formed at 50 °C and broken via retro Diels-Alder reaction at around 120 °C, thus appearing very useful to repair surface scratches or bulk cracks of polymeric materials [16,17,25]. The mechanical properties of these materials can also be tuned by adding fillers within the matrix as demonstrated in earlier works [13,15]. Polyketones functionalized with furan groups and crosslinked with bismaleimide experienced the enhancement of their elastic and loss moduli and softening points thanks to the reinforcement provided by multi-walled carbon nanotubes (MWCNTs). In addition, the percolative networks created by the MWCNTs dispersions within the thermosets allowed for electrically-triggered self-healing phenomena [15]. 

In recent literature, graphene is reported to outperform carbon nanotubes in polymeric nanocomposites in terms of enhanced mechanical [26,27], thermal, and electrical properties [28]. Graphene is one of the most popular nanofiller due to its carbon-like nature, nanometric dimensions and high conjugation of the π electrons. These characteristics render graphene as the most appealing filler in nanocomposites since it exerts its beneficial functions even at very low loading (<2–5 wt%) [29,30]. However, its poor dispersibility in solvents and irreversible aggregation in several media may limit its use [31]. An alternative to graphene is reduced graphene oxide (rGO) which is produced by the chemical or thermal reduction of graphene oxide. The residual functional groups remaining in the rGO structure make its dispersion easier and stable in water and polar organic solvents [32,33,34,35]. Furthermore, they increase the number of effective interactions with polymer matrices improving its homogeneous distribution in the solid host [36,37], leading to the preparation of polymeric nanocomposites [38,39,40], where rGO provides substantial electrical and mechanical properties [41,42,43,44,45]. Gudkov et al. demonstrate that with only 0.6 wt% of rGO dispersed in a polymeric matrix an electrical conductivity of 0.5 S/m is achieved for the nanocomposite [46]. Cheng et al. added rGO as nanofiller into a PVA polymer matrix providing an improvement of the Young’s modulus of about 80%. Despite a good increase in the Young’s modulus, a consequent drop of the elongation at break should be however taken into account [47,48].

**Figure 1:**
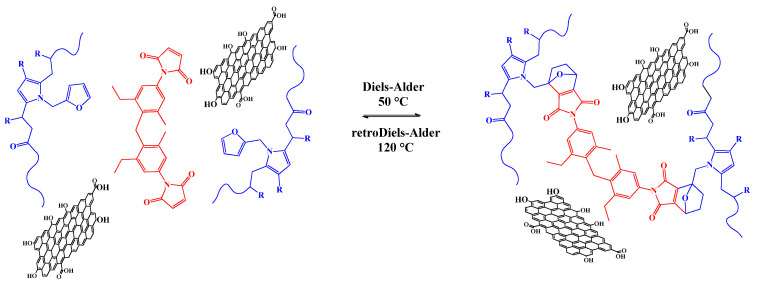
Schematic representation of the formation of the self-healing electrically-conductive nanocomposite based on the functionalized PK (blue), BISM (red) and the rGO (black) nanofiller.

In the present work, we report the synthesis of a self-healing and electrically conductive nanocomposite that displays re-workability and tunable electrical and thermomechanical properties as a function of the rGO content added to the matrix. As starting material, we used polyketone obtained from the copolymerization of 30% ethylene, 70% propylene and CO (PK) and functionalized with furan moieties (PKFU) that act as diene in the reversible Diels-Alder crosslinking process and compatibilizer for the rGO filler [13,15,16]. Moreover, a new type of bismaleimide (BISM) was used as dienophile [13,14,15,16,17] due to its increased miscibility in polyketone that might allow for a faster reaction with PKFU in the bulk (Figure 1). Spectroscopic evidence confirmed the modification of polyketone via the Paal-Knorr reaction and the crosslinking of the final composites. The mechanical properties, recyclability, and reworkability of the final materials were evaluated by thermomechanical analyses. The final morphology of the materials and the dispersion of rGO in the polymeric matrix were characterized by SEM and the modulable OFF-ON electrical conductivity eventually determined as a function of the temperature. 

## 2. Materials and Methods

Furfurylamine (FU ≥ 99%) was purchased from Sigma-Aldrich (Milan, Italy). The amine was freshly distilled before use to remove the oxidized impurities, if any. Bis(3-ethyl-5-methyl-4-maleimidophenyl)methane (BISM abcr 98%, Karlsruhe, Germany), was used as the cross-linking agent. Reduced graphene oxide (rGO) was purchased from Sigma-Aldrich (Milan, Italy), and used without any purification. It is composed by >75% C and <5% N, has a surface area of 103 (m^2^/g) and an electric conductivity of 7111 S/m (compressed powder). N-Methyl-2-pyrrolidone (NMP), deuterated chloroform (CDCl_3_) used for ^1^H-NMR studies, were all purchased from Sigma-Aldrich and used as delivered. Chloroform (CHCl_3_, 99.5%) was purchased from Sigma-Aldrich (Milan, Italy) and used as received. The alternating aliphatic polyketone was synthesized according to the reported procedure [49], using 30% of ethylene and 70% of propylene to obtain the so-called polyketone 30 (PK).

### 2.1. Furfurylamine Functionalization of Alternating Polyketone

The chemical modifications of polyketone (PK) to yield PK with furan groups (PKFU) was carried out in bulk, following the reported procedure [17]. Briefly, 32.50 g of PK was preheated to the liquid state at 110 °C and 14.39 g of FU were added dropwise during 20 min. The reaction proceeded for 4 h and the color of the mixture changed from the typical yellow of the PK to the typical amber-brown of the functionalized polymer due to the presence of the pyrrole units. The resulting polymer was dissolved in 100 mL of chloroform and washed with Milli-Q water to remove the unreacted amine, if any. The washed polymer was dried under air first, and then in a vacuum oven at 50 °C for 48 h, to remove any trace of solvent. The resulting polymer was frozen with liquid nitrogen and crushed to obtain a fine powder. The elemental composition of the samples was analyzed using the Euro EA elemental analyzer. The carbonyl conversion (*C_co_*), i.e., the molar fraction of 1,4-dicarbonyl units reacted via the Paal–Knorr reaction, was calculated on the basis of elemental analysis using the following expression:

(1) $$C_{co}=\frac{y}{y+x}\times 100\%$$

where *x* and *y* are the di-ketone and pyrrolic moles after conversion, respectively. *y* was determined as follows:

(2) $$y=\frac{wt\left(N\right)}{A_{m}\left(N\right)}$$

where *wt*(*N*) are the grams of *N* of the product as determined by elemental analysis and *A*_m_(*N*) is the atomic mass of *N*. *x* was then determined as follows:

(3) $$x=\frac{g_{prod}-y\times M_{w}^{y}}{M_{w}^{pk}}$$

where $g_{prod}$ is the gram of the product, $M_{w}^{y}$ the molecular weight of the pyrrolic unit and $M_{w}^{pk}$ the molecular weight of the 1,4 di-ketone unit. From the ratio between *C_co_* and the corresponding amount in the feed ($C_{co}^{feed}$), the conversion efficiency η con be calculated with the following equation:

(4) $$\eta =\frac{C_{co}}{C_{co}^{feed}}\times 100\%$$

where $C_{co}^{feed}$ corresponds to:

(5) $$C_{co}^{feed}=\frac{Mol_{amine}}{Mol_{d-CO}}\times 100\%$$

where $Mol_{amine}$ are the moles of amine and $Mol_{d-CO}$ the moles of dicarbonyl units in the feed.

### 2.2. One Pot Reaction for the Preparation of the Cross-Linked Nanocomposite

The thermoset nanocomposite was prepared by one-pot solvent-mix containing equimolar amounts of PKFU and BISM (at a furan/maleimide ratio of 1:1) and 1.6, 2.7, 4.3, 5.5 and 7.0 wt% of rGO. The reactants were previously dissolved in NMP (0.87 g of BISM dissolved in 3 mL and 1.00 g of PKFU in 4 mL) and the rGO (respectively 0.03, 0.05, 0.08, 0.103, 0.13 g in 3 mL) tip sonicated (400 W and 24 kHz with UP 400 S probe in titanium with a 3 mm diameter tip and 100 mm length (Hielscher’s H3, Hielscher, Teltow, Germany) for 5 min in NMP to disperse the nanofiller. The reaction mixture was heated up to 80 °C for 24 h under stirring to form the cross-linked network. After cooling to room temperature, the solvent was removed under vacuum at 80 °C for 48 h. The obtained nanocomposite was crushed after freeze drying with liquid nitrogen. The thermoset composed only with PKFU and BISM was synthesized as mentioned above and used as control sample. 

### 2.3. Preparation of Bars by Compression Moulding

Sample bars (approximately 50 mg of sample for a bar of 45 mm long, 5 mm wide and 1 mm thick) were obtained by compression moulding. The samples were moulded at 150 °C at 40 bars for 30 min and then cured at 50 °C in an oven at atmospheric pressure for 24 h. For the preparation of the bars, a FONTIJNE Vlaardingen press (Fontijne presses, Delft, The Netherlands) quipped with a temperature controller was used.

### 2.4. Characterization

ATR–FT–IR spectra were recorded using a Spectrum One instrument (Perkin-Elmer, Waltham, MA, U.S.), within the 4000–650 cm^−1^ and averaged over 32 scans. 

^1^H-NMR spectra were recorded using a Varian Mercury Plus 400 MHz machine (Bruker, Billerica, MA, USA), using CHCl_3_-d as a solvent. 

The elemental composition of the polymer was determined by using an Elementar Vario Micro Cube (Milano, Italy) for nitrogen, carbon and hydrogen.

Microscopic morphology of the composite samples was observed by scanning electron microscopy (SEM) using a Dual Beam FIB/SEM Helios Nano-Lab 600i (FEI, Hillsboro, OR, USA) in a way similar to procedures previously reported [20].

Differential scanning calorimetry (DSC) was carried out using a TA DSC250 system (TA Instruments, New Castle, DE, USA) under N_2_ as described in previous studies [13,15].

Dynamic mechanical thermal analysis (DMTA) measurements were performed using a DMA-8000 machine (Perkin Elmer, Waltham, MA, USA) as reported in previous studies [13,15]. 

The temperature dependent resistivity measurements were performed on square 6 mm × 6 mm samples with 1.05 mm of thickness, connected with two copper electrodes to the opposite edge of the square: the temperature control was obtained by placing the sample on a thin ceramic plate substrate (0.3 mm) equipped with a gold resistance (on the bottom side, not in direct contact with sample) connected to a controllable power supply module. The temperature was monitored with a k-thermocouple brought in contact with the sample; the resistance was continuously measured using a High precision multimeter (Model 187, Fluke, Fluke Corporation, Everett, WA, USA) connected with the copper electrodes. Measurements were performed modulating the temperature by changing the current applied to the ceramic heater and waiting for the temperature stabilization [20]. 

Thermal degradation of the rGO and the derived nanocomposites was analyzed via thermogravimetric analysis (TGA) with a Mettler Toledo TGA/SDTA851 instrument (Mettler Toledo, Columbus, OH, USA) under nitrogen flux (80 mL/min). All samples were tested in agreement with procedures previously reported [48].

Raman spectroscopy has been performed using a Horiba Jobin Yvon Xplora ONE confocal Raman microscope (Horiba Scientific, Horiba Italy, Rome, Italy). The wavelength of the excitation laser was 542 nm and the power of the laser was kept below 1 mW to avoid sample heating [20].

## 3. Results and Discussion

### 3.1. Furfurylamine Functionalization of Alternating Polyketone

**Figure 2:**
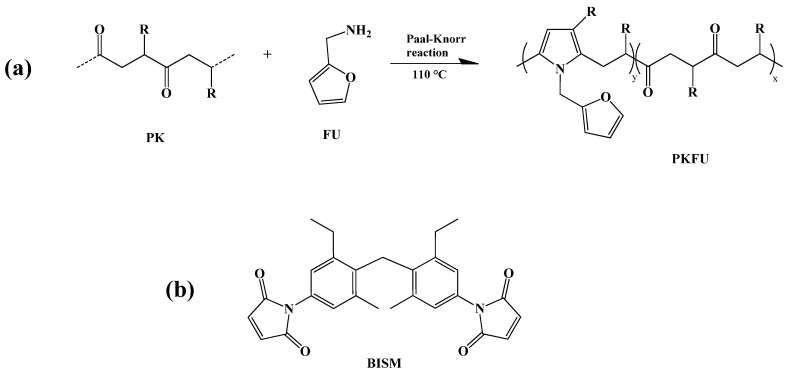
(**a**) Schematic representation of the polyketone modification with furfurylamine and (**b**) the chemical structure of the BISM.

We prepared PKFU by a chemical modification of PK with furfurylamine via the Paal-Knorr reaction (Figure 2a). 

The reaction was carried out in a 0.8 molar ratio between the 1,4-dicarbonyl groups of the PK and the amino groups according to the calculated carbonyl conversion. The functionalization of PK yielded a di-carbonyl conversion (CO %) of 68.3% as determined from elemental analysis.

**Figure 3:**
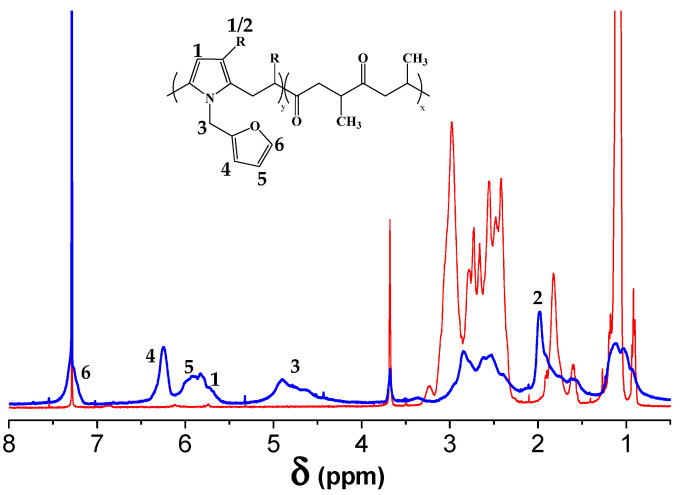
^1^H-NMR spectrum of the pristine polymer (PK) and of the polyketone modified with furfurylamine (PKFU).

We confirmed the successful functionalization of PK by ^1^H-NMR (Figure 3), where the signal at 5.8 and 2.0 ppm were respectively attributed to the hydrogen and to the methyl group of the pyrrole ring formed during the Paal-Knorr process. The peak at 4.9 ppm, attributed to the CH_2_ units connecting the pyrrole ring to the furan one, evidenced the presence of furan as a pendant group. The peaks at 7.3, 6.2 and 5.9 ppm belong to the protons of the furan moieties, as reported earlier [15,18]. Notably, all peaks are attributed to PKFU, thus confirming that no residual amine was present after purification [14,17]. 

**Figure 4:**
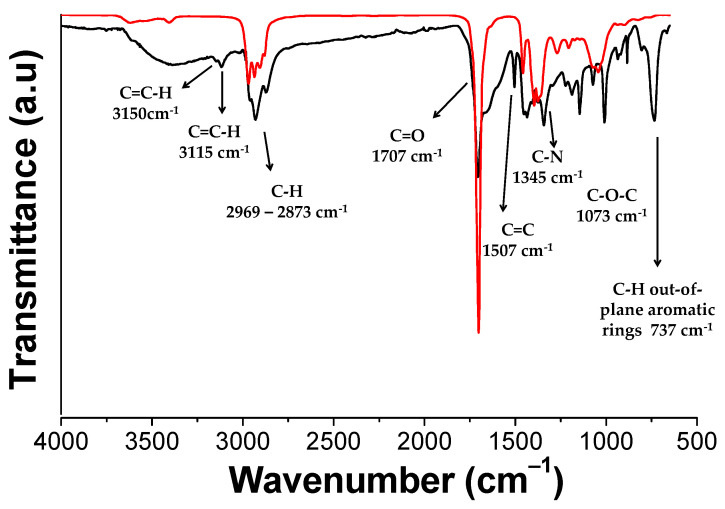
FT-IR spectrum of the pristine polymer (PK, red line) and of the polyketone modified with furfurylamine (PKFU, black line).

Figure 4 shows the ATR-FTIR spectra of PK and PKFU. After the Paal-Knorr reaction occurred, the C=O band centered at 1707 cm^−1^ decreased due to the disappearance of the 1,4-dicarbonyl moieties. The typical peak of the heterocyclic moieties appeared at 1507 cm^−1^ due to the C=C stretching, confirming the presence of both furan and pyrrole groups. The peaks associated to the pyrrole units were found at 3115 cm^−1^ (C=C-H) and at 1345 cm^−1^ (C-N). In addition, we assigned the peaks at 3150 cm^−1^ (C=C-H), at 1073 cm^−1^ (C-O-C), and at 737 cm^−1^ (C-H bonds out-of-plane bending) to the furan pendant group. Finally, the stretching bands of aliphatic C-H of PK backbone and functional groups appeared between 2969 and 2873 cm^−1^ [13].

### 3.2. Preparation and Characterization of PKFU/BISM rGO Nanocomposites

**Figure 5:**
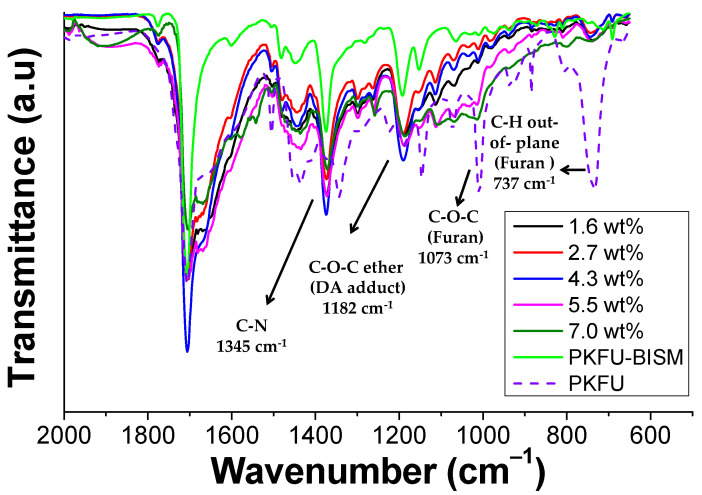
FT-IR spectra of the modified non-cross-linked polymer (PKFU), modified cross-linked polymer (PKFU-BISM), and of the modified cross-linked nanocomposite (PKFU/BISM/rGO).

The bismaleimide (BISM, Figure 2b) was chosen as a cross-linker for various reasons. Notably, the Diels-Alder adduct is reversible, a fundamental feature for imparting thermoplasticity and self-healing characteristics to the material. Moreover, the creation of the network increases the softening point of the PK, which has been calculated to be around 21 °C. The achieved softening temperature depends on the ratio between BISM and the furan pendant groups [16]. Figure 5 shows the ATR-FTIR spectrum of the polymer network before and after crosslinking. The peaks at 1182 and 1378 cm^−1^ were attributed to the C-O-C ether peak of reacted furan and to the stretching of the C-N bond of the maleimide ring, respectively, and confirmed the successful crosslinking between the furan and the bismaleimide moieties. It is worth noting that the intensity of the peaks at 737 cm^−1^ and 1073 cm^−1^ attributed out-of-plane bending C-H and to the C-O-C ether bonds of furan, respectively, decreased due to the formation of the Diels-Alder adduct [17]. 

**Figure 6:**
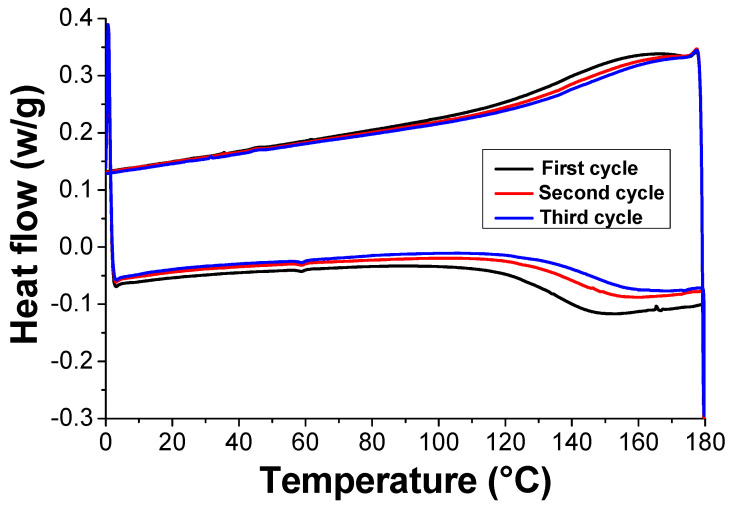
DSC thermogram of the cross-linked nanocomposite containing the 5.5 wt% of rGO.

We characterized all the crosslinked samples by differential scanning calorimetry (DSC) to determine the thermal behavior of the material and the reversibility of the crosslinking process upon three subsequent cycles of heating and cooling. For brevity, we show only the thermogram obtained from the polymer containing 5.5 wt% of nanofiller, while reporting all other results in the supporting information section (Figures S1 and S2). Figure 6 shows the three heating and cooling cycles, all displaying the occurrence of the retro Diels-Alder reaction (endothermic peaks) and the re-formation of the network during cooling. Both broad endothermic and exothermic processes can be found in the range between 120–180 °C. 

In the heating experiments, the peak maximum corresponds to the temperature at which the majority of the Diels-Alder adducts are broken and the area associated to the peak indicates the energy absorbed during the cleavage of the adducts. We calculated the temperature and the energy needed to cleavage the adducts related to the maximum of the peak for each sample (Figure 6 and Figure S1). 

**Figure 7:**
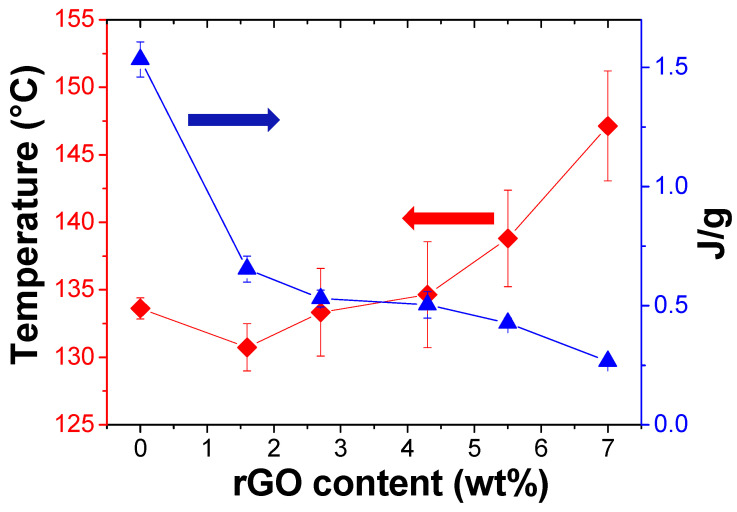
Temperature peak (♦) and enthalpy (▲) where most of the DA adducts are cleaved for all the series of PKFU/BISM/rGO composites at different rGO content.

Notably, as seen in Figure 7 as the temperature increased by more than 10 °C with the filler content, the energy substantially diminished by about 1.25 J/g, thus indicating that rGO affected the thermal stability of the crosslinked system. This effect was already observed in PKFU networks doped with MWCNTs although apparently smaller than that reported here for rGO [15]. 

**Figure 8:**
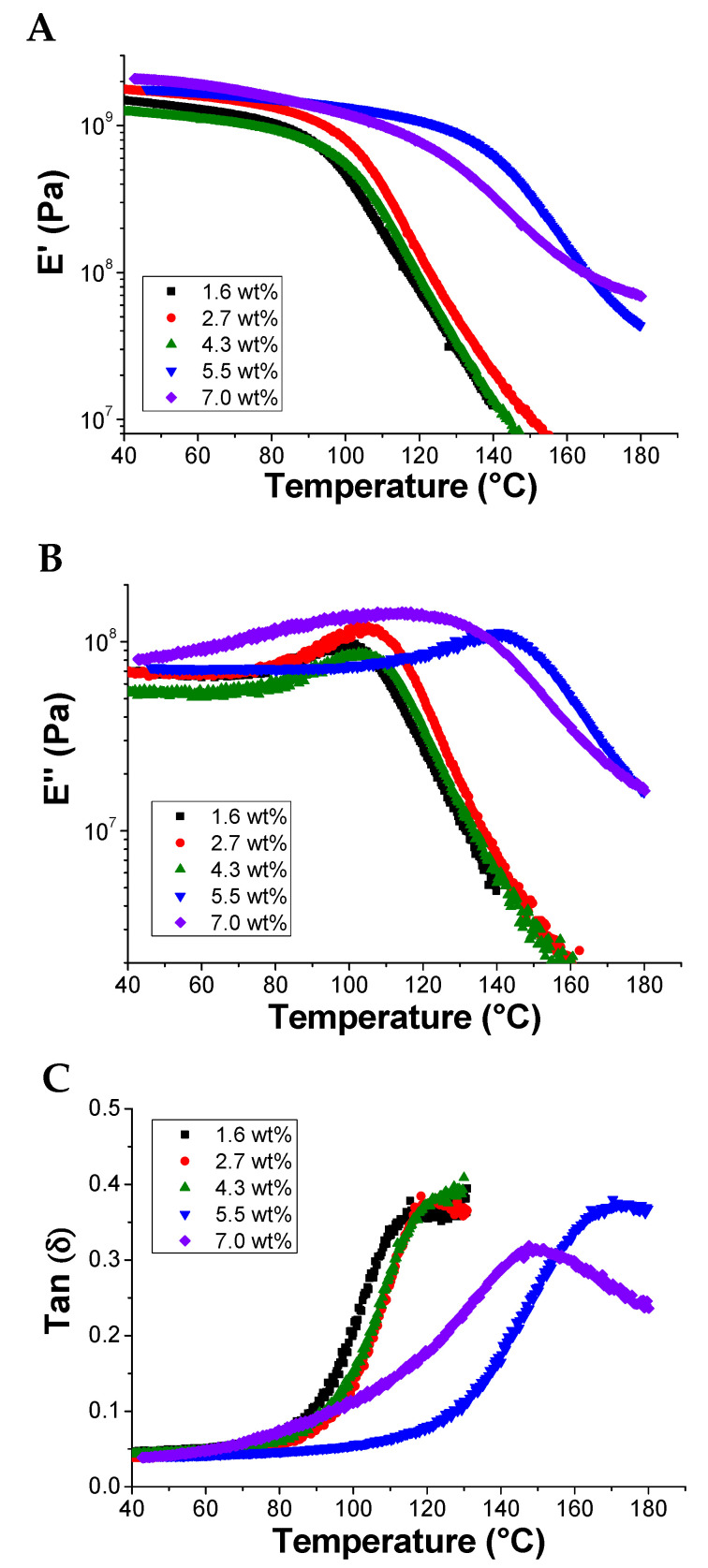
Storage modulus E′ (**A**), loss modulus E″ (**B**) and tan δ (**C**) of the rGO/PKFUBISM with different amounts of rGO as a function of the temperature.

The mechanical behavior of the composites as a function of temperature was investigated by dynamic mechanical thermal analysis (DMTA) in the range from 40 °C to 180 °C (Figure 8). The bar samples for the DMTA analysis were obtained by compression molding of grinded crosslinked composites at 150 °C and 40 bar for 30 min. This method promotes the rDA mechanism and consequently the de-crosslinking of the thermoset nanocomposite. This behavior evidences the reversible and recyclable nature of these composite materials. The variation in storage modulus (E′), loss modulus (E″) and tan δ (softening point) were measured as a function of the rGO content. All samples show an initial slow decrease of the storage modulus and a corresponding increase of the loss modulus until a maximum value is reached which corresponds to the softening point of the material. After this point, a faster decrease of both moduli occurs due to the higher softening rate of the material. Notably, both loss and elastic moduli and softening point rise substantially for rGO contents higher than 5 wt%, thus confirming the reinforcement characteristics of the graphitic fillers in polymers [15]. Remarkably, the softening point increased from ~120 °C for the composite with a 1.6–4.3 wt% of rGO to values higher than 140 °C at loadings larger than 5.5 wt%. This effect could be possibly attributed to the interfacial interactions between graphitic filler and the polymer. rGO concentrations higher than 7 wt% seem to show minor reinforcements effects possibly due to less effective phase dispersion of the filler in the polymer matrix at the highest content.

**Figure 9:**
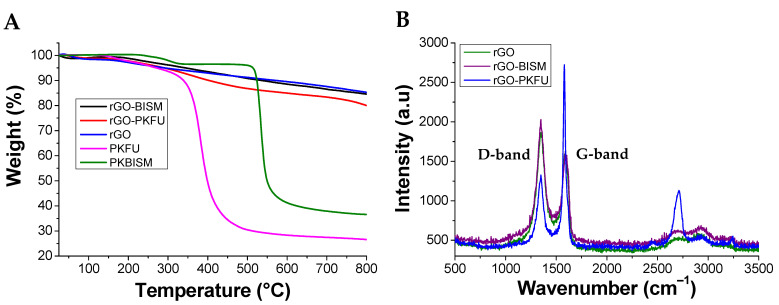
TGA (**A**) and Raman analysis (**B**) of rGO mixed with BISM and PKFU.

In order to understand the nature of the interactions between rGO and the network components, rGO was mixed with PKFU or BISM under the same conditions carried out for the formation of the rGO/PKFUBISM composite. After mixing, the recovered rGO was washed with chloroform several times to remove the non-interacting PKFU or BISM. We then evaluated the amount of PKFU or BISM interacting with rGO by thermogravimetric analysis. The experiments were performed from 25 °C to 800 °C under nitrogen atmosphere (Figure 9A). PKFU experiences a thermal degradation starting about 350 °C and showed a weight loss of 70%. rGO begin to degrade at about 200 °C with a gradual rate and it did not display a plateau even at 800 °C. On the other hand, BISM showed two degradation stages at 250 °C and around 500 °C with a total weight loss of 60%. rGO-PKFU also shows two degradation stages one at 200 °C due to the thermal degradation of the oxygen-functional groups of rGO [48] and a second one around 350 °C due to the degradation of PKFU grafted onto rGO. This sample showed an overall weight loss of 5.3% with respect to the pristine rGO at 800 °C. Conversely, rGO-BISM showed an identical thermal degradation than pristine rGO and an overall weigh loss of only 0.7 with respect to pristine rGO at 800 °C. Therefore, PKFU seems to interact better with the surface of rGO with respect to the neat BISM. 

The same samples were also characterized in terms of Raman spectroscopy, since it gives useful insights for the structure determination of graphitic materials (Figure 9B). The D band (1340 cm^−1^) and G band (1580 cm^−1^) are typical peaks of the rGO Raman spectrum [50,51]. The G-band indicates the planar vibration of sp^2^ carbon atoms forming the graphitic structure whereas the D-band is associated to the scattering from defects that break the fundamental symmetry of the graphene sheet [52,53]. The peak centered at 2900 cm^−1^ is the combination of the first overtone of the D band (2D band) and the D+G band. The ratio of D and G bands peak intensities (I_D_/I_G_) are widely used as standard index to identify defects on rGO after its functionalization. In this respect, the functionalization of rGO with PKFU inverts the intensities of the D and G-band and the I_D_/I_G_ ratio of rGO and rGO-PKFU changed from 1.15 to 0.73, respectively. This result has already been published for functionalized carbon nanotubes with polyketones bearing furan groups through the Diels-Alder reaction [13]. In addition, rGO-PKFU shows a red-shift and sharpening of the G-Band in comparison to the pristine rGO. Kudin et al., addressed the sharpening and shifting back to graphitic position (shift to lower energies) of the G-Band in rGO after the heating treatment of graphene oxide [54] to the restoration of the rGO aromaticity in the defect regions of the graphene layer [55]. Overall, it seems that the effective interaction between PKFU and rGO could be attributed by a covalent bonding between the two materials and possibly caused by the Diels-Alder between the diene PKFU and the rGO dienophile. Future work will be addressed to investigate the reliability of the proposed mechanism further. Conversely, the functionalization of rGO with BISM slightly modified the intensities of the D and G-band, thus confirming the poor interaction between the crosslinker and the graphitic filler. 

**Figure 10:**
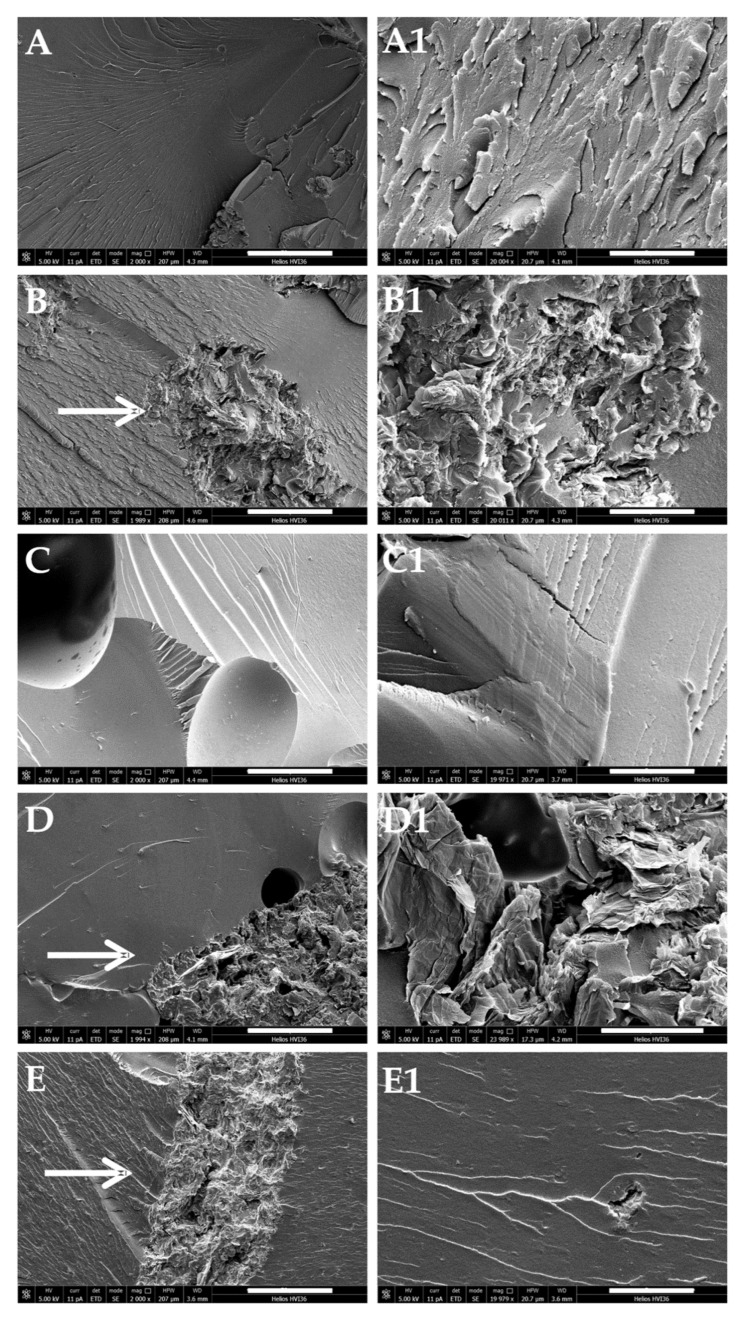
Scanning Electron Microscopy (SEM) micrographs at different magnification of the nanocomposite composed by PKFU/BISM and rGO at different weight percentage. (**A**,**A1**) 1.6 wt%, (**B**,**B1**) 2.7 wt%, (**C**,**C1**) 4.3 wt%, (**D**,**D1**) 5.5 wt%, and (**E**,**E1**) 7 wt%. Left pictures scale bar 50 μm, right pictures scale bar 5 μm.

We also evaluated the morphology of the composite material and the dispersion degree of rGO by SEM microscopy. Figure 10 shows the SEM images of the cross-sectional area of all the investigated nanocomposites and at different magnifications. In general, all samples appear as a smooth polymer matrix that contains assemblies of rGO randomly distributed in the form of interacting flakes with the polymer matrix but apparently distributed as isolated clusters. This effect appeared well evident in samples containing the highest amount of rGO (Figure 10D,E for 5.5 wt% and 7 wt% of rGO loading). While on one side the interfacial interactions between the polymer matrix and the rGO appeared confirmed, on the other these were not totally effective in providing a homogeneously distributed filler content within the crosslinked PKFU/BISM network.

### 3.3. Electrical Conductivity Properties of PKFU/BISM rGO Nanocomposites

**Figure 11:**
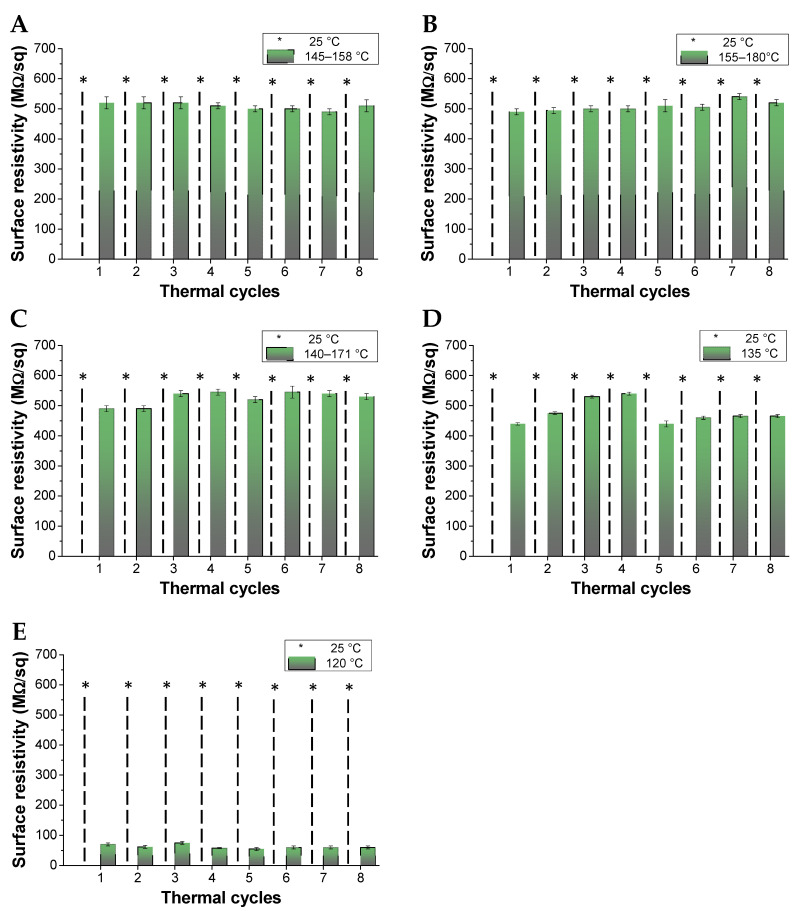
Surface resistivity of the nanocomposite composed by PKFU/BISM and rGO at different weight percentage. (**A**) 1.6 wt%, (**B**) 2.7 wt%, (**C**) 4.3 wt%, (**D**) 5.5 wt%, and (**E**) 7 wt%. * ≥500 MΩ/sq. Sample thickness of 1.05 mm.

We then investigated the electric behavior of the nanocomposites in terms of surface resistivity and as a function of the rGO content. Contrary to our expectations, no samples were able to conduct electricity at room temperature (Figure 11) even at the highest rGO content and also after the annealing process carried out at temperature higher than the corresponding softening points. These results could be possibly attributed to the generation of ineffective percolation pathways of conductive rGO assemblies within the polymer network and potentially attributed to the phase dispersion behavior evidenced by the SEM analysis (Figure 10). Conversely, all the investigated nanocomposites started to conduct electricity at temperatures higher than 100 °C and with absolute surface resistivity values depending on the rGO concentration. The nanocomposite with rGO content of 1.6 wt%, 2.6 wt%, 4.3 wt% showed surface resistivity around 500 MΩ/sq at temperatures higher than 150 °C (Figure 11A–C) and after each cycle the temperature fluctuated considerably, suggesting that the nanocomposite is still close to the percolation threshold. Increasing the rGO content, the temperature threshold decreased to 135 °C and below 100 °C for the 5.5 wt% and 7.0 wt% of filler, respectively. Notably, a substantial decrease in the surface resistivity to below 100 MΩ/sq occurred at the highest 7 wt% doping amount of the rGO filler. Such OFF-ON reversible and temperature depending conductive behavior can be due to a combination of effects: (a) during annealing, the polymer becomes softer and the rGO particles are keen to generate effective percolation pathways thanks to their higher mobility within the polymer network; (b) rGO is a typical semiconductor, whose resistance decreases with temperature increase [56,57,58], thus allowing the polymer network to conduct electricity. 

### 3.4. Self-Healing and Mechanical Properties of the Nanocomposite

**Figure 12:**
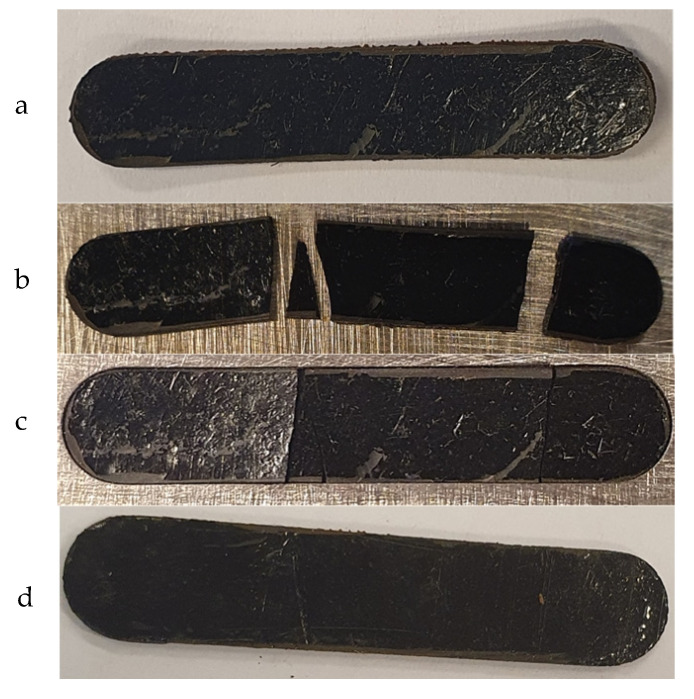
Bar of PKFUBISM containing the 2.7 wt% of rGO after molding (**a**), breaking (**b**), inside the mold (**c**) and after self-healing (**d**). Bar size: 45 mm long, 5 mm wide and 1 mm thick.

When testing the recyclability or the self-healing of a new material, mechanical properties are the biggest and most important issue. The recovery of the mechanical properties of the thermoset after the self-healing is essential because otherwise the material cannot be used to any further extent, even if all the other properties are recovered. To test the self-healing effect on the mechanical properties of our material, a bar of the polymer was prepared by compression molding, and DMTA analysis was performed. Then, the bar was broken as reported in Figure 12 and remolded using the same condition used before. Once healed, a second DMTA was collected on the cured sample (Figure 12d). In Figure 12 the pristine bar is shown (a) as well as the broken one (b,c). We noticed that after remolding (d) the bar recovered its original shape without any cracks, thus confirming the self-healing characteristic of the material for this first attempt. 

**Figure 13:**
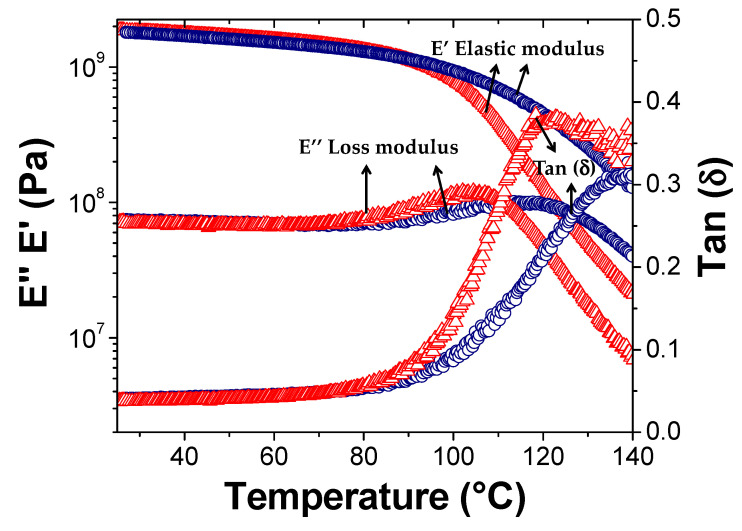
DMTA result of storage modulus (E′), loss modulus (E″) and tan (δ) of PKFUBISM/rGO 2.7% after molding (△) and after the self-healing (○).

The previous result was confirmed by the DMTA characterization. Storage and loss moduli of the thermoset before and after the self-healing show the same values until 100 °C, while exhibiting an opposite behavior at higher temperature (Figure 13). This behavior was reflected on the softening point of the material, the Tan (δ), which increased from ~120 °C to ~140 °C in the healed sample. The enhanced rigidity of the composite material after healing might be addressed to the presence of rGO, whose interfacial interactions with the PKFU macromolecules become more effective as also recently observed in similar samples containing MWCNTs [15,42,44,47,59]. 

## 4. Conclusions

In this work, we have prepared a new polymer composite characterized by electrical conductivity and self-healing characteristics triggered by temperature variations. The starting material used for this purpose is a polyketone, obtained by the polymerization of CO, ethylene and propylene. The polyketone was chemically modified by Paal-Knorr reaction using furfurylamine to introduce a furan pendant group onto the backbone of the polymer. The modification of the polyketone with furfurylamine successfully proceeded with a very high yield of about 85%, as confirmed by elemental analysis, FT-IR and ^1^H-NMR. Nanocomposites were then prepared by solution mixing using reduced graphene oxide as a nanofiller and BISM as crosslinker to confer self-healing properties. After cross-linking via the formation of a Diels-Alder adduct between the furan moieties linked to PK and the BISM a cross-linked network was obtained, characterized by a reversible character being destroyed at 120 °C and reformed at 50 °C as confirmed by ATR-FTIR and DSC. The increased softening temperature of the cross-linked polymer was confirmed by DMTA which showed a maximum in the tan δ values of ~120 °C for the polymer containing 1.6, 2.7, 4.3 wt% of nanofiller and 170 °C for the polymer containing 5.5 wt%. All the nanocomposites showed a storage modulus higher than 10^6^ Pa for all the investigated temperature, confirming the robustness of the polymer also at high temperature. The electrically conductive behavior of the crosslinked nanocomposite occurred only at temperatures higher than 100 °C with an OFF-ON behavior possibly addressed to the semiconducting features of rGO distributed in the polymer network in the form apparently isolated clusters as evidenced by SEM microscopy. The self-healing ability of the nanocomposite was confirmed by testing the recovery of the mechanical properties after the breaking and reprocessing processes of the polymer network. Overall, the conductive self-healing nanocomposite reported in this work can be useful for several applications where conductivity is required only at temperature higher than a certain threshold thus suggesting the development of electronic switches triggered by temperature. Notably, this smart material is also cost-effective thanks to the favorable selection of low-cost materials and the small amount of required rGO.

## Data Availability

The data presented in this study are available on request from the corresponding author.

## Supplementary Materials

The following are available online at https://www.mdpi.com/2073-4360/13/3/339/s1. Figure S1. DSC thermal cycles of PKFU/BM/rGO nanocomposites at different rGO content (wt%). Figure S2. TGA of the PKFU/BM/rGO nanocomposites at different rGO content (wt%).
