# Supplementary material for: Thermally Switchable Electrically Conductive Thermoset rGO/PK Self-Healing Composites
Source: Polymers (Basel). 2021 Jan 21;13(3):339. doi: 10.3390/polym13030339 (PMC7865638; doi:10.3390/polym13030339)
Supplement: Supplementary file 1 [file polymers-13-00339-s001.pdf]

polymers

MDPI

Supplementary Materials

Thermally Switchable Electrically Conductive Thermoset rGO/PK Self-Healing Composites

Esteban Araya-Hermosilla [1], Alice Giannetti [2], Guilherme Macedo R. Lima[3], Francesco Picchioni[3], Virgilio Mattoli[1], Ranjita K. Bose[3], and Andrea Pucci[2,4*]

[1] Center for Micro-BioRobotics, Istituto Italiano di Tecnologia Viale Rinaldo Piaggio 34, Pontedera (PI) 56025, Italy; esteban.araya@iit.it (E.A.-H.); virgilio.mattoli@iit.it (V.M.)
[2] Dipartimento di Chimica e Chimica Industriale, Università di Pisa, Via Moruzzi 13, 56124 Pisa, Italy.
[3] Department of Chemical Product Engineering, ENTEG, University of Groningen, Nijenborgh 4, 9747AG Groningen, The Netherlands; f.picchioni@rug.nl (F.P); g.de.macedo.rooweder.lima@rug.nl (G.M.R.L); r.k.bose@rug.nl (R.K.B)
[4] CISUP, Centro per l'Integrazione della Strumentazione dell'Università di Pisa, Lungarno Pacinotti 43, 56126 Pisa, Italy
* Correspondence: andrea.pucci@unipi.it (A.P.).
Tel.: +39 0502219270 (A.P.).

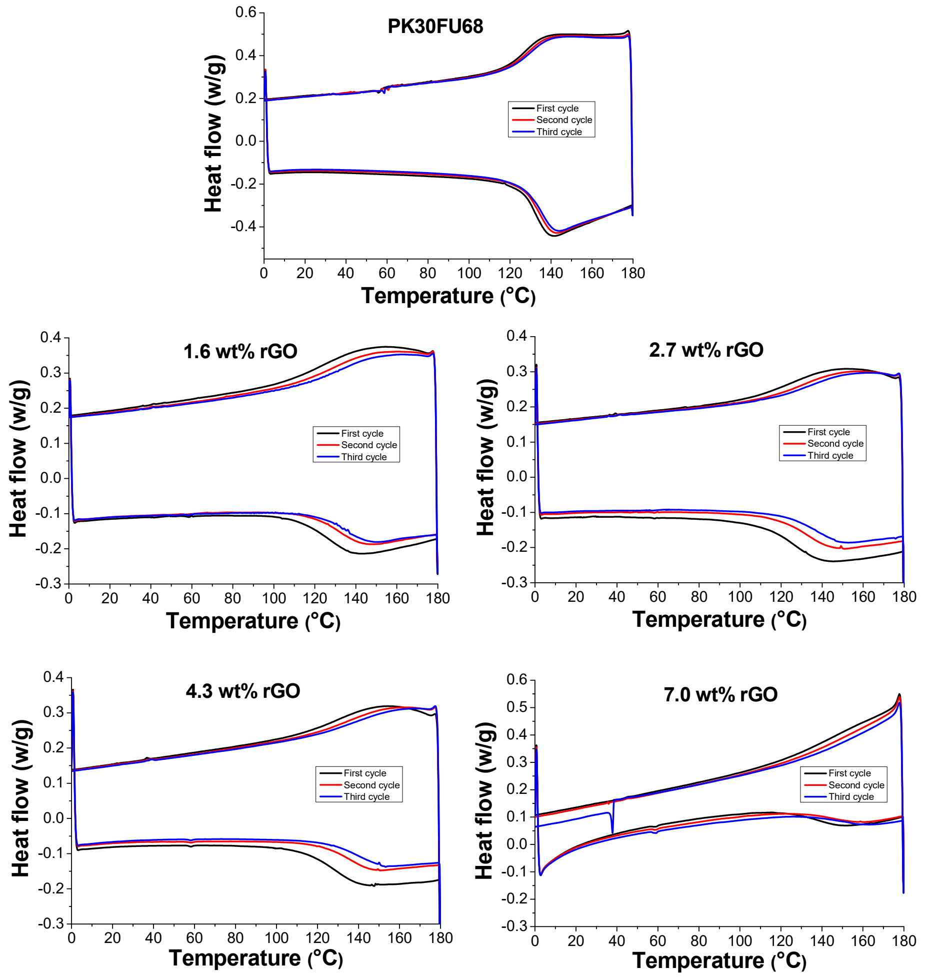

Figure S1. DSC thermal cycles of PKFU/BM/rGO nanocomposites at different rGO content (wt%)

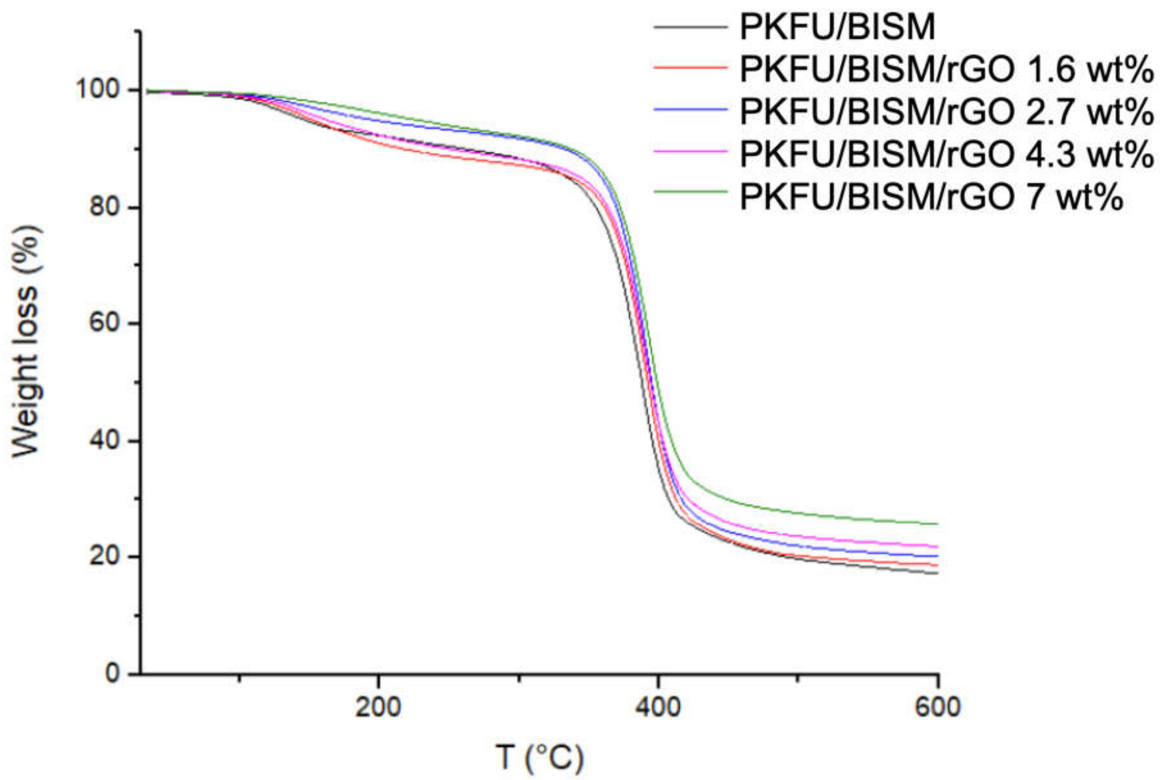

Figure S2. TGA curves of PKFU/BM/rGO nanocomposites at different rGO content (wt%)
